# A Case of Complicated Pneumonia Caused by Klebsiella ozaenae

**DOI:** 10.7759/cureus.23001

**Published:** 2022-03-09

**Authors:** Takayuki Tachibana, Naoto Mouri, Chiaki Sano, Ryuichi Ohta

**Affiliations:** 1 Family Medicine, Shimane University Medical School, Izumo, JPN; 2 Community Care, Unnan City Hospital, Unnan, JPN; 3 Community Medicine Management, Faculty of Medicine, Shimane University, Izumo, JPN; 4 Communiy Care, Unnan City Hospital, Unnan, JPN

**Keywords:** general medicine, rural hospital, pneumonia, klebsiella, immunocompromised, bacteremia, antibiotic resistance

## Abstract

*Klebsiella ozaenae*, a subtype of *K. pneumonia*,* *causes chronic upper respiratory tract infections, such as rhinitis and rhinoscleroma, and can also cause lethal infections. We report the case of a patient who developed pneumonia caused by *K. ozaenae*. An 87-year-old man presented to our hospital with fever and chills. Physical examination revealed no findings other than bilateral crackles in the lower lung fields. Chest computed tomography (CT) showed infiltrative shadows in the lower left lung field. Moreover, *K. ozaenae* was detected in blood cultures. Based on the examination results, including radiography and blood culture, the patient was diagnosed with pneumonia caused by *K. ozaenae*. On admission, the patient was treated with intravenous ceftriaxone (CTRX), but he did not recover. After determining the antibiotic susceptibility of *K. ozaenae*, we stopped administering CTRX and started ampicillin/sulbactam (ABPC/SBT) treatment for two weeks. During the ABPC/SBT administration, a second chest CT showed a new infiltrative shadow in the upper left lung field. Despite these findings, the patient was discharged from the hospital as his vital signs were stable and his general condition was good. After two weeks of ABPC/SBT treatment, the patient was switched to minocycline and followed up. Although infections caused by *K. ozaenae* are rare, they can be life-threatening. *K. ozaenae* identification in a patient’s blood culture indicates a potentially impaired immune system, prompting physicians to evaluate the patient’s immune system.

## Introduction

The genus *Klebsiella *consists of non-motile, facultatively anaerobic, gram-negative rods. The genus is a member of the Enterobacteriaceae family and belongs to the normal flora of the human mouth and intestine [1]. Patients with alcoholism are more likely to have established *Klebsiella *in the nasopharyngeal bacterial flora as chronic alcohol intake contributes to malnutrition, leading to the breakdown of local protective barriers in the respiratory tract [2,3]. When doctors diagnose and treat pneumonia, it is important that physicians ask the patient about their alcohol intake.

Of the pathogenic *Klebsiella *species, *Klebsiella pneumoniae* is the most prevalent. However, *K. ozaenae* is the causative pathogen of some rare diseases, including primary atrophic rhinitis [4]. Although *K. ozaenae* is a rare human pathogen that seldom causes serious infection [5,6], it can cause fatal diseases, such as sepsis, meningitis, and cerebral abscess [5-9]. Risk factors for *K. ozaenae* bacteremia include chronic rhinitis, old age, prior antibiotic usage, immunosuppression, malignancy, alcohol abuse, and diabetes mellitus [5]. *K. ozaenae* is rarely isolated and, to our knowledge, has not previously been reported to cause pneumonia. Here, we describe a case in which *K. ozaenae *caused pneumonia and sepsis in a patient without immunosuppression or diabetes mellitus.

## Case presentation

A 65-year-old man with a history of chronic kidney disease, hypertension, and stable prostate cancer five years after radiation therapy presented to our hospital with a fever and chills. Two days prior to admission, he had undergone dermatological surgery to remove cysts on the abdominal wall, and cephalexin was administered. No abnormality was found at the surgical site. He had phlegm without coughing. He did not complain of any other symptoms, including respiratory difficulties, headache, vomiting, or dysuria. On examination, he had a temperature of 39.4°C, a heart rate of 97 beats/minute, a respiratory rate of 24 breaths/minute, and an oxygen saturation of 96% breathing room air. Auscultation revealed bilateral crackles in the lower lung fields. No other abnormalities were noted. Blood and sputum cultures were then performed to identify potential infectious pathogens. The laboratory test results showed a white blood cell count of 8,400 cells/µL with neutrophil dominance, hemoglobin, blood urea nitrogen, and creatinine levels of 13.2 g/dL, 25.6 mg/dL, and 1.64 mg/dL, respectively (Table 1).

**Table 1:** Laboratory findings on admission.

Laboratory parameter	Day 1	Reference range
White blood cell (×10^3^/μL)	8.4	3.5–9.1
Red blood cell (×10^6^/μL)	4.03	3.76–5.50
Hemoglobin (g/dL)	13.2	11.3–15.2
Hematocrit (%)	39	33.4–44.9
Mean corpuscular volume (fL)	96.8	79.0–100.0
Platelet (×10^4^/μL)	13.1	13.0–36.9
Neutrophil (%)	79	44.0–72.0
Lymphocyte (%)	12	18.0–59.0
Monocyte (%)	8.8	0.0–12.0
Eosinophil (%)	0	0.0–10.0
Basophil (%)	0.2	0.0–3.0
Aspartate aminotransferase (U/L)	25	8–38
Alanine aminotransferase (U/L)	18	4–43
Alkaline phosphatase (U/L)	127	106–322
Gamma-glutamyl transpeptidase (U/L)	25	0–48
Lactate dehydrogenase (U/L)	222	121–245
Total protein (g/dL)	7.8	6.5–8.3
Albumin (g/dL)	39	3.8–5.3
Blood sugar (mg/dL)	128	60–109
Blood urea nitrogen (mg/dL)	25.6	8–20
Creatinine (mg/dL)	1.62	0.40–1.10
Serum Na (mEq/L)	133	135–150
Serum K (mEq/L)	4	3.5–5.3
Serum Cl (mEq/L)	93	98–110
Estimated glomerular filtration rate (mL/minute/1.73 m^2^)	31.7	<60.0

**Figure 1:**
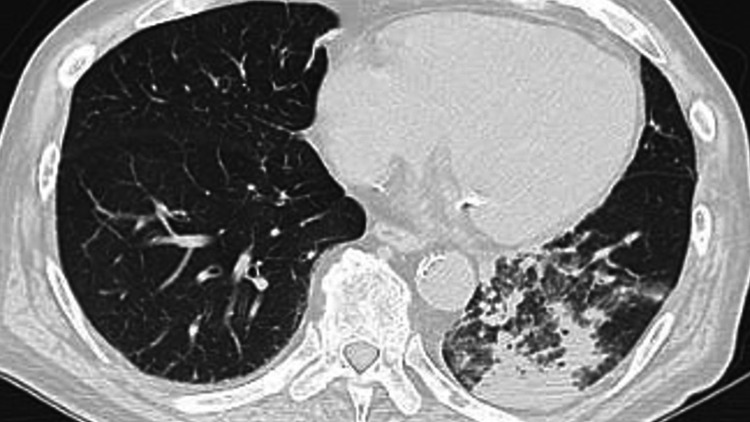
Initial chest computed tomography scan showing opacity in the left lower lung zone at admission.

Chest computed tomography (CT) revealed an opacity in the left lower lung lobe (Figure 1).

We diagnosed pneumonia based on the clinical findings. The CURB65 score was two points, and the patient was treated with ceftriaxone (CTRX, 2 g/day).

*K. ozaenae* was identified on blood culture, and *Streptococcus mitis* and *Neisseria *species were identified in sputum cultures four days after admission. The antibiotic susceptibility test revealed that the *K. ozaenae* isolate was resistant to CTRX (Table 2).

**Table 2:** Antimicrobial susceptibility of Klebsiella ozaenae and Klebsiella aerogenes. MIC: minimal inhibitory concentration; R: resistant; I: intermediate; S: susceptible

Antimicrobial agents	*Klebsiella ozaenae*	*Klebsiella aerogenes*	MIC (μg/mL)
Ampicillin	I	R	16
Piperacillin	S	S	≤16
Cefazolin	S	R	≤2
Cefotiam	I	R	16
Ceftriaxone	R	R	>2
Ceftazidime	R	S	>16
Cefepime	R	S	<16
Cefaclor	S	R	≤8
Cefmetazole	R	R	>32
Latamoxef		S	>8
Imipenem	S	S	≤1
Meropenem	S	S	≤1
Doripenem	S	S	≤1
Aztreonam	R	S	>16
Sulbactam/ampicillin	S	S	≤8
Tazobactam/piperacillin	S	S	
Gentamicin	S	S	
Tobramycin	S	S	
Amikacin	S	S	
Levofloxacin	S	S	
Ciprofloxacin	S	S	
Sulfamethoxazole- trimethoprim	S	S	
Fosfomycin	R	I	
Colistin	R		

**Figure 2:**
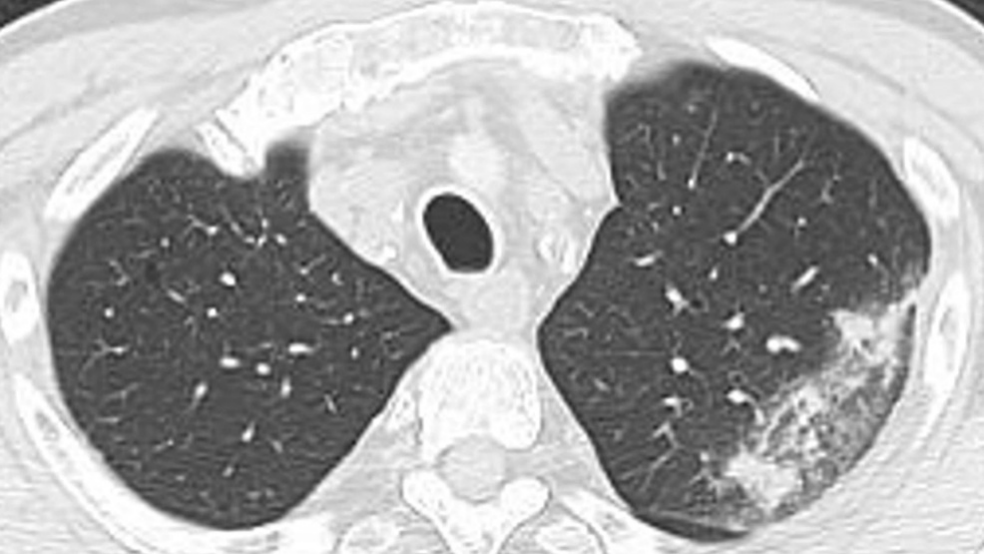
Second chest computed tomography scan revealing that opacity in the lower left lung lobe had improved but a new opacity had appeared in the left upper lung lobe.

Therefore, the patient’s treatment was switched to ampicillin/sulbactam (ABPC/SBT). During ABPC/SBT treatment, the patient relapsed with fever but recovered, and 12 days after admission, a follow-up chest CT revealed that the opacity in the lower left lung lobe had improved, but a new opacity had developed in the left upper lung lobe (Figure 2).

**Figure 3:**
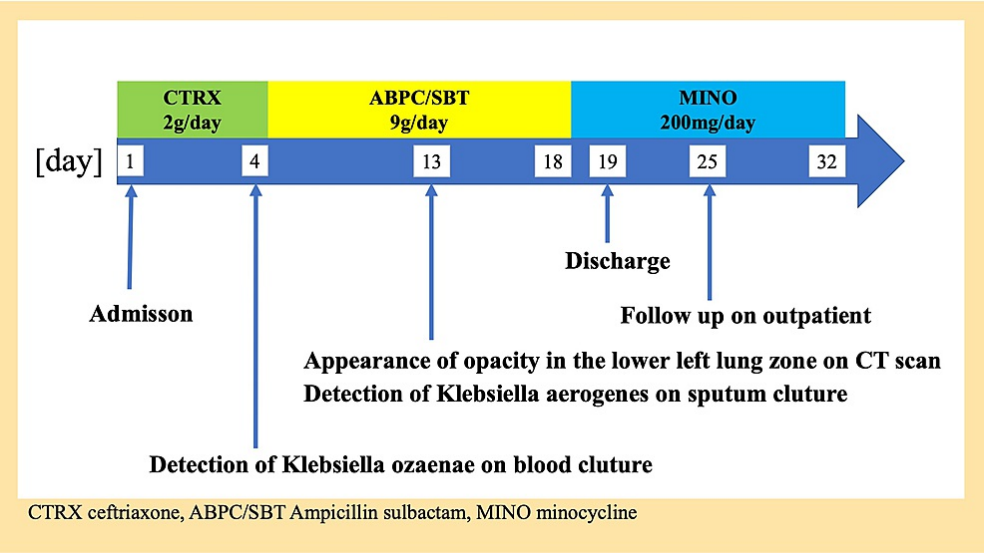
Clinical course of the case.

However, *K. ozaenae* was not identified in an additional blood culture and sputum, and *K. aerogenes*, which was sensitive to ABPC/SBT and minocycline (MINO), was detected only in the sputum culture (Table 2). Because the opacities in the lower left lung lobe improved but remained, the glycoprotein Krebs von den Lungen-6 (KL-6) was measured to assess the presence of organizing pneumonia. ABPC/SBT was continued because KL-6 levels were within the normal range. After two weeks of ABPC/SBT treatment, the patient was in good general condition. Therefore, he was switched from ABPC/SBT to MINO and then discharged (Figure 3).

Six days after discharge, he was followed up, and his condition was stable. There were no abnormalities of note on examination, including auscultation. The patient was treated with MINO for two weeks.

## Discussion

The patient presented with pneumonia and bacteremia caused by *K. ozaenae* and was treated at our hospital. However, during treatment, he developed pneumonia in different lung areas caused by *K. aerogenes*, which differs from *K. ozaenae*. *K. aerogenes* and *K. ozaenae* are unusual human pathogens that rarely cause serious infections in healthy individuals. Immune system dysfunction was suspected in the patient, and *K. ozaenae* detection in blood cultures confirmed that the patient’s immune function could be impaired. We suggest that it is important to evaluate the immune function of a patient along with the treatment of the disease.

This case presentation of *K. ozaenae* as pneumonia is rare. *K. ozaenae* is the causative agent of non-fatal diseases, such as chronic rhinitis and rhinoscleroma [4]. Although infections caused by *K. ozaenae* are rare, life-threatening infections have been reported [5], and *K. ozaenae* can cause fatal diseases, such as sepsis, meningitis, and cerebral abscess [5-9]. Reported risk factors for *K. ozaenae* bacteremia include immunosuppression and diabetes mellitus [5].

The diagnosis and treatment of pneumonia caused by *K. ozaenae* can be challenging. Our patient was diagnosed with pneumonia and bacteremia caused by *K. ozaenae*. We reached this diagnosis for three reasons. First, auscultation revealed crackles in the bilateral lower lung field areas, and chest CT revealed opacity in the left lower lung zone. Second, no other abnormalities were observed. Third, although *K. ozaenae* was not detected in the sputum, *K. ozaenae* was identified in the blood culture. This is why sputum classified into Group 3 by the Geckler classification was collected from the patient’s mouth where *Streptococcus mitis* and *Neisseria *species are normal commensal flora. In addition, despite being treated with CTRX, the patient did not recover. However, he recovered after switching the antibiotic treatment regimen to ABPC/SBT, an antibiotic to which *K. ozaenae* was susceptible.

The selection of antibiotics to treat *K. ozaenae* should be based on sensitivity, organ penetration, and host immunity. Although the patient improved during ABPC/SBT treatment, he had a relapse of fever, and a chest CT revealed a new opacity in the left upper lung zone. The relapse of symptoms was hypothesized to be due to the onset of pneumonia caused by *K. aerogenes* based on sputum culture results and his medical history including reduced kidney function. Reduced kidney function has been reported to be associated with an increased risk of serious infection in older individuals [10]. However, *K. ozaenae* might have caused pneumonia in this area either because ABPC/SBT was only partially effective, or because an abscess had formed; therefore, the ABPC/SBT treatment was considered ineffective. The causes of a partial response to antibiotic therapy consist of three aspects: the antibiotic, the target pathogen, and the patient’s body system. These aspects include the bacterial status, inoculum size, antibiotic concentrations, serum effects, and host-gut microbiome interactions [11]. However, we ruled out these hypotheses because *K. ozaenae* was not detected in the later cultures and because the second chest CT revealed that opacity in the lower left lung zone had improved. *K. aerogenes* infection can cause pneumonia, immunodeficiency, and nosocomial infections.

Patients’ immune function should be considered if *K. ozaenae* is detected on blood culture. Despite presenting with pneumonia and bacteremia, our patient did not have evidence of an inflammatory reaction, such as an elevated white blood cell count. These findings suggest that he had a dysfunctional immune system [12]. Moreover, compared with immunologically healthy individuals, patients with immune dysfunction are susceptible to infection by organisms of low native virulence or experience increased severity of common infections [13].

## Conclusions

When *K. ozaenae* is identified in blood culture, it indicates that a patient’s immune system may be impaired. Thus, physicians should evaluate the patient’s immune system. Because *K. ozaenae* can cause life-threatening infections in immunosuppressed patients, careful follow-up is required. *K. ozaenae* not only causes non-lethal infections, such as rhinitis and rhinoscleroma, but can also cause life-threatening infections, such as pneumonia and sepsis, particularly in immunocompromised patients or patients with diabetes mellitus. When *K. ozaenae* is detected in blood cultures, it is important to treat not only the local infection site but also assess the functioning of the patient’s immune system.
